# DMSG-SLAM: Cascaded Semantic and Geometric Filtering for RGB-D Tracking and Mapping in Dynamic Environments

**DOI:** 10.3390/s26123634

**Published:** 2026-06-07

**Authors:** Beicheng Li, Enhui Zheng, Huailiang Wang, Yuhao Geng, Qiming Hu, Xuxu Qi

**Affiliations:** School of Mechanical and Electrical Engineering, China Jiliang University, Hangzhou 310018, China; p24010854052@cjlu.edu.cn (B.L.); p24010854108@cjlu.edu.cn (H.W.); p24010854029@cjlu.edu.cn (Y.G.); s24010811023@cjlu.edu.cn (X.Q.)

**Keywords:** simultaneous localization and mapping, dynamic environments, masking, geometric constraints, visual sensors

## Abstract

Traditional visual SLAM systems often suffer from localization drift in dynamic environments due to interference from moving objects. Although semantic segmentation and depth-based masking methods have improved performance, they may still suffer from boundary under-segmentation and missed detections due to truncation of dynamic objects. To address these challenges, we propose a cascaded framework, DMSG-SLAM, a cascaded visual SLAM system that fuses Depth-Mask, Semantic information and Geometry constraints for dynamic environments. A lightweight object detection network, combined with depth consistency, is first employed to generate instance-like masks for preliminary dynamic feature removal. Then, a rotation-aware local epipolar geometric filtering mechanism is introduced to suppress residual features near object boundaries and mitigate perceptual blind spots caused by occlusion or truncation. Within potential dynamic regions, the epipolar threshold is adaptively switched according to the estimated inter-frame rotation to provide a more conservative filtering effect under challenging motion conditions. In addition, a TSDF-based dense volumetric map is incorporated to reconstruct more consistent surfaces. Experiments on highly dynamic sequences from the TUM RGB-D dataset indicate that DMSG-SLAM achieves competitive accuracy in dynamic environments, with localization performance improving by up to 90% compared to ORB-SLAM2.

## 1. Introduction

Simultaneous localization and mapping is a core technique for localization and environment perception [1]. In the absence of prior knowledge of the environment, it leverages data acquired from one or more sensors to estimate the sensor pose with centimeter-level accuracy while simultaneously constructing a map of the surroundings. SLAM has been widely applied in robotics, as well as in augmented reality and virtual reality applications. Conventional visual SLAM methods, such as ORB-SLAM2 [2], are typically built upon the assumption of a static environment. While they can achieve high accuracy under such conditions, real-world scenarios often contain dynamic objects that interfere with the SLAM pipeline, leading to degraded localization performance [3]. Consequently, effectively detecting and removing dynamic objects has become a critical challenge in visual SLAM [4].

Early efforts addressed this problem using purely geometric approaches. By exploiting rigid-body geometry or motion consistency, outliers that violate geometric constraints can be identified and removed [5]. However, geometry-only methods lack semantic understanding of the scene and typically rely on fixed thresholds that are uniformly applied to all feature points, limiting their adaptability [6].

With the rapid advancement of deep learning, researchers have increasingly incorporated semantic information into visual SLAM systems for dynamic object removal. To some extent, learning-based approaches offer clear advantages: object detection [7,8] and semantic segmentation [9,10] can accurately recognize objects that have been seen during training, thereby enabling more precise elimination of dynamic features associated with these objects. However, purely semantic methods also exhibit notable limitations. They are generally unable to handle previously previously unseen dynamic objects. Moreover, approaches that rely solely on object detection often remove dynamic features using coarse bounding boxes, which may inadvertently discard a large number of useful static features. When the bounding boxes are overly large, the number of retained feature points may become insufficient, leading to tracking failure [11]. In addition, employing fine-grained segmentation methods significantly increases the computational burden, thereby reducing system efficiency [12].

Recent studies have attempted to combine deep learning with depth-based approaches by leveraging both semantic and depth information to generate instance-like masks. By integrating object detection with depth-connected component analysis, instance-level masks can be efficiently produced, significantly reducing the computational cost of running object detection on every frame and enabling real-time performance in RGB-D dynamic SLAM systems. Despite the effectiveness of such “detection + depth” mask generation strategies in typical scenarios, they still face several challenges under complex dynamic interactions and extreme motion conditions [13]: First, coarse boundary segmentation remains a critical issue. Due to the measurement noise of depth sensors near object boundaries, masks generated solely from depth information are often unable to accurately align with dynamic contours. Such under-segmentation allows dynamic points along object edges to leak into the static set, thereby contaminating pose estimation. Second, object detection networks are prone to missed detections. When dynamic objects appear near image boundaries or are heavily occluded, detection failures may occur, leaving residual dynamic feature points unfiltered and consequently introducing localization drift. Finally, spatiotemporal instability arises under complex motion. In scenarios involving large camera rotations or rapid object movements, conventional epipolar geometric constraints tend to become less reliable, leading to the misclassification of a substantial number of static background points [14].

**Figure 1:**
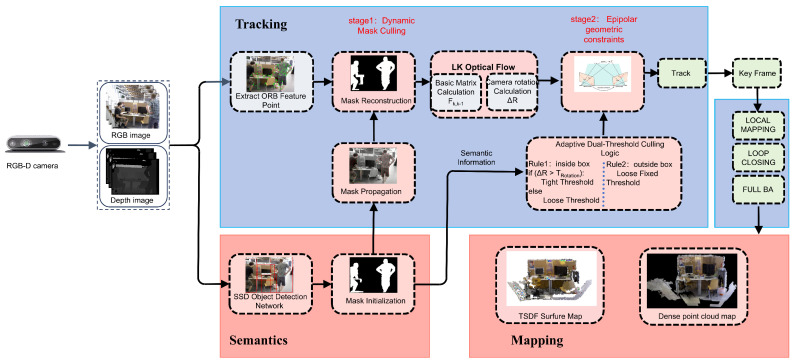
Architecture of the DMSG-SLAM system. The blue region denotes the ORB-SLAM2 baseline, while the red region highlights the proposed components.

To address these issues, we design a dynamic SLAM framework following a “mask-first, geometry-refinement” strategy. First, a lightweight object detector combined with depth information is employed to rapidly generate instance-like masks, providing a relatively clean initialization for pose estimation. Subsequently, a rotation-adaptive multi-level epipolar geometric constraint is introduced as a secondary refinement stage. This mechanism not only removes residual features near mask boundaries caused by under-segmentation, but also serves as a complementary fallback to alleviate localization drift caused by detection blind spots or unseen dynamic objects. Through this cascaded filtering scheme, the system improves its handling of complex dynamic environments. In addition, both a global point cloud map and a TSDF-based dense volumetric map are constructed. The overall framework of DMSG-SLAM is illustrated in Figure 1.

The main contributions of this work are summarized as follows.

(1) We propose a cascaded dynamic visual SLAM system based on the RGB-D mode of ORB-SLAM2, termed DMSG-SLAM. Compared with the baseline method, the proposed system achieves an improvement of approximately 90% in localization accuracy on highly dynamic sequences from the TUM dataset. Meanwhile, a TSDF-based mapping module is integrated into the backend, enabling real-time reconstruction of dense surface maps.
(2) A cascaded dynamic feature culling strategy is introduced to improve system accuracy in complex environments. A semantic thread first generates depth-aware masks to suppress feature points associated with potential dynamic objects. To further handle residual dynamic interference, a rotation-aware local geometric filtering mechanism is introduced within potential dynamic regions, where the epipolar threshold is adaptively switched according to the estimated inter-frame rotation. This design helps suppress residual dynamic features near object boundaries and missed moving objects while maintaining a practical balance between tracking accuracy and efficiency.
(3) A dedicated surface reconstruction thread based on an octree-based truncated signed distance function is introduced. Guided by the refined poses from the dynamic filtering module, this component incrementally fuses observations to generate a smooth and dense 3D surface map in real time, providing a an environmental representation for downstream tasks such as path planning, obstacle avoidance, and scene interaction.

The remainder of this paper is organized as follows. Section 2 reviews the related work. Section 3 presents the proposed DMSG-SLAM framework, including the overall pipeline and its key components. Section 4 reports the experimental results and analysis. Finally, Section 5 concludes the paper and discusses future research directions.

## 2. Related Works

### 2.1. Geometry-Based Dynamic SLAM

Geometry-based dynamic SLAM approaches primarily remove dynamic features by constructing mathematical models such as multi-view geometry, epipolar constraints, or reprojection consistency, without relying on predefined semantic labels [15]. Specifically, Yang et al. [16] combine the Euclidean distance of feature points with epipolar geometry to achieve a two-stage dynamic feature filtering strategy. Kundu et al. [17] introduce an odometry-guided “optical flow boundary” on top of epipolar constraints to address detection degradation when the camera and objects move in the same direction. Nguyen et al. [18] separate dynamic and static regions by measuring the deviation between observed optical flow and a theoretically predicted one-dimensional trajectory. Zhang et al. [19] perform dynamic segmentation by subtracting ego-motion-induced optical flow from the total scene flow and analyzing the residual in the 2D image domain. Furthermore, Zou and Tan, in CoSLAM [20], distinguish dynamic from static points based on the consistency of reprojection errors and enable motion trajectory reconstruction. Although geometric constraints can mitigate certain dynamic disturbances, methods relying on handcrafted feature matching and geometric consistency checks remain limited in complex environments. Under severe occlusions or abrupt illumination variations, feature matching often degrades, resulting in increased computational burden and ultimately causing localization drift [21]. In recent years, researchers have gradually incorporated semantic information into geometric constraints. RTS-SLAM [22] employs a trajectory consistency-driven multi-constraint strategy to eliminate dynamic feature outliers. It first integrates semantic information and epipolar geometric constraints for coarse filtering of dynamic points, and then uses trajectory consistency constraints to remove residual dynamic features by analyzing the alignment between feature 3D motion and camera ego-motion. However, such methods still rely on post-verification after feature matching and cannot actively isolate dynamic regions in advance, and therefore remain limited under complex dynamic conditions.

### 2.2. Semantic and Mask-Based Dynamic SLAM

To balance real-time performance and estimation accuracy in dynamic environments, recent studies have increasingly explored the integration of lightweight semantic priors with depth information to generate dynamic masks. For instance, NGD-SLAM [23] adopts a mask propagation mechanism, leveraging YOLO-based detection boxes together with depth cues to achieve efficient object segmentation while reducing computational overhead under limited resources. However, approaches that rely primarily on semantic priors still face notable challenges in complex scenarios. As demonstrated in GeneA-SLAM2 [24], the generalization capability of object detectors can degrade under conditions such as rapid camera rotation or high-speed object motion. This often leads to missed detections, resulting in incomplete coverage of dynamic regions and leaving residual interfering features along object boundaries. Although existing methods refine the results using constraints such as depth variance, purely semantic-driven strategies still exhibit inherent perceptual blind spots when dynamic objects are truncated at image boundaries.

To address these limitations, this paper proposes a feature filtering framework that integrates semantic depth-based masking with epipolar geometric verification, aiming to exploit the complementarity between semantic cues and geometric consistency in dynamic environments. Unlike approaches that rely primarily on increasingly complex mask refinement, the proposed method emphasizes logical decoupling and functional complementarity. For regions within prior detection boxes, the proposed approach builds upon the mask generation strategy and further introduces epipolar geometric verification as an auxiliary filtering mechanism. To alleviate the under-segmentation issue near semantic boundaries, a rotation-aware local threshold-switching strategy is introduced: when the estimated inter-frame rotation exceeds a predefined threshold, a stricter epipolar threshold is applied within these potential dynamic regions; otherwise, a relatively looser threshold is used. For regions outside the prior detection boxes, a fixed epipolar geometric verification mechanism is incorporated to suppress residual dynamic features from previously unseen moving objects as well as low-quality correspondences. In this way, the geometric stage serves as a complementary safeguard in regions that are insufficiently covered by semantic priors.

### 2.3. Dense 3D Mapping in Dynamic Environments

Early RGB-D reconstruction methods were predominantly developed under the assumption of static environments and could achieving highly accurate 3D mapping under ideal conditions [25]. KinectFusion [26] pioneered the use of truncated signed distance functions for voxel-based implicit surface representation, enabling continuous fusion of depth data to effectively suppress sensor noise. Subsequently, ElasticFusion [27] introduced a lightweight surfel-based map representation and discarded the traditional pose graph formulation, instead performing loop closure optimization directly on the global map via a non-rigid deformation model. However, in the presence of dynamic disturbances, these geometric assumptions often break down, leading to tracking failures and degraded map quality. To address the limitations of static-scene assumptions, MaskFusion [28] proposed a decoupled reconstruction paradigm that integrates instance-level semantic masks with geometric segmentation, maintaining separate surfel maps for the static background and each independently moving object. Similarly, DynaSLAM [29] combines multi-view geometric constraints with deep learning techniques to explicitly remove potentially dynamic pixels during mapping, relying exclusively on highly reliable static features to reconstruct clean background maps and employing background inpainting to compensate for occluded regions. To further improve dense mapping in dynamic scenes, RTS-SLAM provides a dense mapping scheme based on global point cloud sparsification and key-region refinement, which reduces memory consumption while preserving important scene structures.

Despite these advances in dynamic environments, such methods typically incur substantial computational overhead. To preserve real-time performance, the proposed approach avoids explicit background inpainting. Instead, it utilizes image and depth information from keyframes outside potential dynamic regions, together with pose estimates from the front-end, to incrementally construct a dense and clean 3D map of the static scene within the mapping thread.

## 3. System Overview

In this section, the implementation details of the proposed DMSG-SLAM system are described. First, the overall framework and fundamental workflow of the system are presented. Next, the employed object detection method and its role are introduced. Then, the generation of dynamic masks via a mask propagation strategy is briefly described. Subsequently, the principles of dynamic feature removal based on epipolar constraints, along with the proposed improvements, are explained. Finally, the construction of the dense point cloud map and the TSDF-based dense volumetric map are introduced.

### 3.1. System Framework

The proposed DMSG-SLAM system is built upon the classical visual SLAM framework ORB-SLAM2. In addition to the original tracking, local mapping, and loop closure threads, two parallel threads, namely a semantic detection thread and a dense mapping thread, are incorporated. The overall system architecture is illustrated in Figure 1. Specifically, the proposed method extends the RGB-D mode of ORB-SLAM2. RGB and depth images are acquired from an RGB-D camera, aligned frame by frame, and then fed into both the tracking and semantic detection threads. In the semantic thread, objects are first identified using an object detection network to obtain candidate dynamic regions. These regions are further refined using depth consistency to generate instance-level masks. The resulting dynamic masks are then transmitted to the tracking thread for feature filtering, while the associated semantic information is preserved for subsequent processing. In the tracking thread, ORB feature points are extracted from the input frames while the masks are being generated. A first-stage dynamic feature removal is performed using the masks to eliminate features associated with potential dynamic objects. Subsequently, the remaining features are further refined through a second-stage filtering process that integrates semantic information with epipolar geometric constraints. This step removes residual dynamic features and low-quality points both inside and outside the detected regions. Meanwhile, an adaptive threshold-switching mechanism is employed to balance outlier rejection and static background feature retention under varying motion conditions, thereby ensuring reliable pose estimation and stable mapping. In the dense mapping thread, depth images are used as the primary geometric source. Combined with keyframe poses for coordinate transformation, global point clouds and a TSDF-based dense volumetric map are incrementally constructed through filtering and fusion.

### 3.2. Object Detection Method

Conventional visual SLAM front-ends cannot explicitly distinguish between dynamic and static objects, while purely geometric approaches often struggle to achieve effective separation within limited computational time.

To address this limitation, an object detection module is incorporated into the front-end, enabling the system to leverage semantic cues from the environment. To satisfy the stringent requirements of real-time performance and computational efficiency, a lightweight SSD [30] detector deployed on the NCNN [31] inference framework is adopted as the semantic perception module. The model is trained on the PASCAL VOC 2007 dataset [32] and is capable of recognizing 20 common object categories. For more effective downstream processing, these categories are further classified into a priori dynamic objects (e.g., humans, birds, and dogs) and a priori static objects (e.g., sofas, televisions, and dining tables). By trading off pixel-level segmentation precision for computational efficiency, the proposed detection module provides coarse yet effective semantic priors, which serve as the foundation for subsequent mask generation and geometric verification.

### 3.3. Dynamic Mask Generation

**Figure 2:**
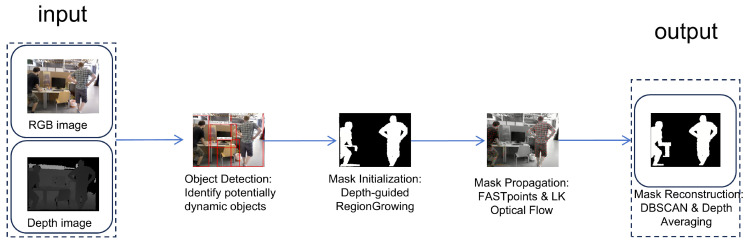
Dynamic mask generation process.

After obtaining reliable object bounding boxes from the NCNN-based detector, the system further extracts more precise pixel-level masks for dynamic feature removal. Drawing inspiration from the concept of cross-frame mask propagation in previous works, this algorithm employs a lightweight mask generation mechanism. By using the object bounding boxes output by the detection framework as prior guidance, the system constructs a “detection–tracking–reconstruction” mask processing workflow, as shown in Figure 2. The specific workflow is as follows:

The first step involves the initialisation of a prior mask based on depth consistency. The system utilizes the SSD detector to obtain 2D bounding boxes of potential dynamic objects, denoted as B=[x,y,w,h]. Since these bounding boxes typically include a significant amount of background pixels, a depth-guided region growing strategy is introduced to refine the mask. Specifically, leveraging the continuity of the depth map, a connected-component labelling algorithm is applied to cluster pixels within the bounding box. Let p=(u,v) denote a pixel in the image coordinate system, and let D(p) represent its depth value. By computing the median valid depth $d_{c}$ within the central region of the bounding box, the initial mask $M_{\mathrm{init}}$ is extracted according to the following criterion, where valid depth denotes reliable depth measurements after eliminating invalid zero values and out-of-range noisy data, and $d_{c}$ is the median depth value calculated in the central area of the detection bounding box:

(1) $$M_{\mathrm{init}}\left(p\right)=\left\{\begin{array}{cc} 1, & \mathrm{if}\;p\in B\wedge |D\left(p\right)-d_{c}|\;<\tau_{d}\\ 0, & \mathrm{otherwise} \end{array}\right..$$

where $\tau_{d}$ denotes the predefined depth tolerance threshold, empirically set to $\tau_{d}\;=\;0.33\;m$. This formulation leverages depth consistency to suppress background interference with significant depth deviations within the bounding box, thereby extracting the effective core region of the target object.

The second step involves inter-frame tracking based on sparse feature point associations. To reduce the significant computational overhead caused by performing object detection on every frame, temporal consistency is exploited to propagate masks across frames. Specifically, within the valid region covered by the initial mask $M_{\mathrm{init}}$, a set of feature points $P_{t}=\left\{p_{1},p_{2},\ldots ,p_{n}\right\}$ is extracted using a grid-based sampling strategy, where the grid resolution dynamically adjusts to the spatial dimensions of the bounding box.

During tracking between consecutive frames, the process is based on the brightness constancy assumption, which states that pixel intensity remains approximately unchanged over a short time interval:

(2) $$I_{t}\left(p\right)=I_{t+1}\left(p+\Delta p\right).$$

Here, $I_{t}\left(p\right)$ denotes the image intensity at time *t*, *p* represents the pixel coordinate in the image plane, and Δp is the pixel displacement vector between adjacent frames.

The Lucas-Kanade optical flow method [33] is employed to perform a first-order Taylor expansion around each feature point, enabling the estimation of the optimal displacement vector Δp:

(3) $$\nabla I{\left(p\right)}^{T}\Delta p+\frac{\partial I(p)}{\partial t}=0.$$

Here, ∇I(p) denotes the spatial image gradient at pixel *p*, and $\frac{\partial I(p)}{\partial t}$ is the temporal derivative of image intensity. This linear approximation is valid provided that the inter-frame pixel displacement is small enough. Meanwhile, the structure tensor established by image gradients needs to be invertible, which requires sufficient texture information in local regions to obtain stable and unique optical flow solutions.

By solving this formulation, the system efficiently propagates the dynamic feature points from time *t* to t+1, yielding the tracked feature set $P_{t+1}={\left\{p_{i}+\Delta p_{i}\right\}}_{i=1}^{n}$. In this way, the mask is transferred across frames with low latency, ensuring temporal consistency.

The third step involves mask reconstruction based on a density clustering algorithm. To address trajectory drift caused by occlusion or rapid motion during optical-flow tracking, the DBSCAN algorithm [34] is introduced to enforce spatiotemporal consistency on the predicted feature points. For any point *p* in the tracked set, its ε-neighborhood is defined as:

(4) $$N_{\epsilon }\left(p\right)=\left\{q\in P_{t+1}|{∥p-q∥}_{2}\leq \epsilon \right\}.$$

Here, $N_{\epsilon }\left(p\right)$ denotes the ε-neighborhood of point *p*, *q* represents an arbitrary feature point in the tracked feature set $P_{t+1}$, $P_{t+1}$ denotes the set of tracked feature points at time t+1, ${∥p-q∥}_{2}$ refers to the Euclidean distance between point *p* and point *q*, and ϵ is the neighborhood radius, empirically set to ϵ=50 pixels. The algorithm partitions the point set into independent motion entities based on spatial density constraints, while automatically identifying and removing outliers that do not conform to the dominant motion patterns.

Finally, the refined inlier set is combined with the depth distribution of the current frame to dynamically reconstruct the mask for each frame, denoted as $M_{\mathrm{final}}$:

(5) $$M_{\mathrm{final}}\left(p\right)=\left\{\begin{array}{cc} 1, & \mathrm{if}\;p\in R\left(T\right)\;\mathrm{and}\;|D_{t+1}\left(p\right)-d_{T}|\;<\tau_{d}\\ 0, & \mathrm{otherwise} \end{array}\right..$$

where R(T) denotes the refined inlier set, $D_{t+1}\left(p\right)$ represents the depth value of pixel *p* in the current frame, $d_{T}$ is the average depth of the inlier set, and $\tau_{d}$ is the depth tolerance defined above. This “detection–tracking–reconstruction” cyclic mechanism ensures that even when semantic detection temporarily fails due to viewpoint changes or motion blur, the system can still rely on the mask prior from the previous frame for continuous forward prediction, thereby maintaining the continuity and completeness of dynamic object removal.

Although the above temporal propagation-based masking strategy is effective in most conventional dynamic scenarios, it still faces challenges under extreme motion conditions. As reported in studies such as GeneA-SLAM2, semantic masks may occasionally exhibit partial failure or incomplete coverage in such cases. This can result in a small number of dynamic feature points leaking into the static background, thereby introducing additional drift in pose estimation.

### 3.4. Rotation-Adaptive Semantic–Geometric Constraints

To mitigate the residual dynamic features that may remain after semantic masking, a rotation-adaptive epipolar geometric constraint is further incorporated as a complementary filtering stage. This mechanism introduces additional geometric verification based on motion consistency. Specifically, within the semantic prior regions, when relatively large camera rotation is observed, the system adaptively tightens the epipolar constraint threshold, thereby enforcing more conservative feature selection. In contrast, for regions outside the prior boxes, a relatively relaxed fixed threshold is applied as a coarse fallback mechanism.

The detailed procedure is as follows. First, the system performs a refined estimation of the fundamental matrix using semantic priors. Feature correspondences between the previous frame $F_{k-1}$ and the current frame $F_{k}$ are tracked using the Lucas-Kanade optical flow method. Based on the object detection results, the potential dynamic region $B_{k}$ in the current frame is obtained, and only feature correspondences outside $B_{k}$ are retained to construct a purely static point set $x_{static}$:

(6) $$x_{static}=\left\{\left(p_{k-1}^{i},p_{k}^{i}\right)|p_{k}^{i}\notin B_{k}\right\}.$$

Here, $p_{k-1}^{i}$ and $p_{k}^{i}$ are paired homogeneous feature points between adjacent frames. Subsequently, the fundamental matrix $F_{k,k-1}$ is estimated using the static feature set $x_{static}$ in conjunction with the RANSAC algorithm. The estimated fundamental matrix effectively encodes the camera motion, including rotation and translation components, and provides the basis for subsequent epipolar constraint computation. It should be noted that the estimation of the fundamental matrix may become unstable in degenerate cases, such as pure or near-pure rotational motion, low-parallax motion, planar scenes, or situations where dynamic correspondences dominate the feature matches. Therefore, in the proposed framework, geometric verification is not used as an isolated decision criterion, but rather as part of a cascaded semantic-geometric filtering strategy guided by semantic priors and complemented by a feature-retention rollback mechanism to avoid excessive feature rejection and maintain tracking stability.

**Figure 3:**
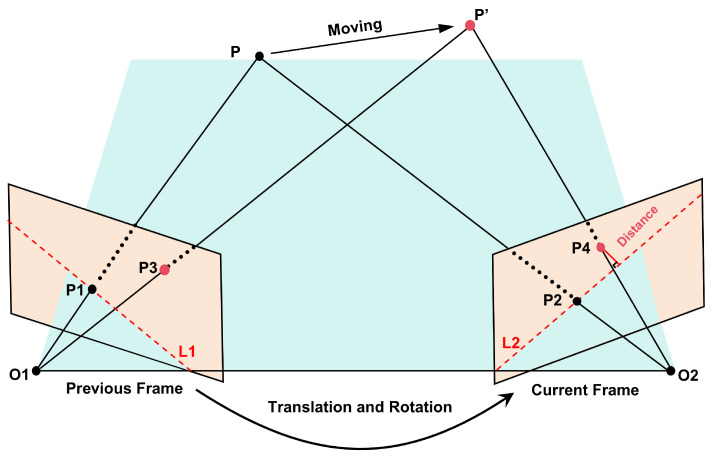
Epipolar geometry constraints.

Once a reasonably accurate fundamental matrix has been obtained, the system performs consistency checks on the remaining feature points using multi-view geometric constraints. The geometric schematic is shown in Figure 3. The relevant symbols are defined as follows:

Here, $O_{1}$ and $O_{2}$ denote the camera centers of two consecutive frames. *P* represents the 3D position of a feature point at time k−1, while $P^{\prime }$ denotes its true position after motion at time *k*. $P_{1}$ and $P_{2}$ are the corresponding projections of *P* in the previous and current frames, respectively. $L_{1}$ and $L_{2}$ denote the epipolar lines in the two consecutive frames.

(7) $$P_{1}=\left[x_{1},y_{1},1\right],\;P_{2}=\left[x_{2},y_{2},1\right].$$

Subsequently, the epipolar line $L_{2}$ in the current frame is computed using the estimated fundamental matrix $F_{k,k-1}$.

(8) $$L_{2}=FP_{1}=F\left[\begin{array}{c} x_{1}\\ y_{1}\\ 1 \end{array}\right]=\left[\begin{array}{c} X\\ Y\\ Z \end{array}\right].$$

where *X*, *Y*, and *Z* denote the components of the epipolar line vector. According to [17], the epipolar constraint corresponding to Figure 3 can be expressed as:

(9) $$P_{2}^{T}FP_{1}=P_{2}^{T}L_{2}=0.$$

Then, the distance between the point $P_{i}$ in the current frame and its corresponding epipolar line is is taken as the deviation distance, denoted by *D*:

(10) $$D=\frac{\left|P_{2}^{T}FP_{1}\right|}{\sqrt{X^{2}+Y^{2}}}.$$

From the above formulation, it can be observed that, under ideal conditions, the deviation distance *D* of static feature points should be zero. However, in real-world scenarios, noise is inevitable, and thus the deviation distance of static points is typically non-zero but remains below a predefined threshold. As depicted in Figure 3, the motion of point *P* to $P^{\prime }$ induces a deviation at $P_{4}$. Once this deviation exceeds the predefined threshold, $P_{4}$ is identified as a dynamic point and excluded from further processing.

A key limitation of epipolar geometry arises under pure rotation or relatively large rotational motion, where the constraint may become less reliable. During large-angle rotations, the fundamental matrix can become ill-conditioned, causing the epipolar distances of true static background points to increase. As a result, a fixed rejection threshold may incorrectly classify these informative static features as dynamic outliers, leading to tracking failure. To alleviate this issue, we introduce a motion-aware adaptive thresholding strategy. By leveraging the constant-velocity motion model in the front-end, the system continuously estimates the inter-frame rotational magnitude θ. The rotation angle is derived from the trace of the predicted rotation matrix *R*:

(11) $$\theta =\arccos\left(\frac{\mathrm{Tr}(R)-1}{2}\right).$$

Here, Tr(R) denotes the trace of the rotation matrix *R*. It should be noted that due to the lag of the constant velocity model, the θ used here is not an accurate physical measurement, but a soft indicator for evaluating the degree of rotational intensity.

Based on the above principles, the system adopts the following threshold strategy for feature points in the image plane.

(12) $$\tau =\left\{\begin{array}{cc} \tau_{out}, & p\notin B_{k}\\ \tau_{loose}, & p\in B_{k}\;\mathrm{and}\;\theta \leq \theta_{th}\\ \tau_{strict}, & p\in B_{k}\;\mathrm{and}\;\theta >\theta_{th} \end{array}\right..$$

Here, τ is the adaptive threshold, and $\theta_{th}$ represents the preset rotation threshold. For feature points outside the potential dynamic target boxes, a fixed and relatively loose threshold is adopted to filter out residual dynamic features that may be missed by the detection network. For feature points inside the potential dynamic target boxes, a relatively loose threshold is used when the estimated inter-frame rotation remains below a predefined threshold, so as to preserve locally static structures. When the estimated inter-frame rotation exceeds this threshold, the system switches to a stricter geometric criterion to suppress dynamic feature leakage and low-quality correspondences. This adaptive mechanism is designed as a conservative filtering strategy within semantically indicated high-risk regions, balancing local static feature preservation with the need to reduce dynamic contamination under challenging motion conditions.

### 3.5. Map Creation

**Figure 4:**
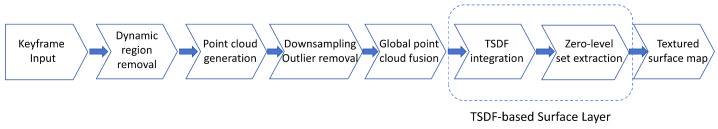
Overview of the TSDF-based 3D textured surface reconstruction workflow in dynamic environments.

The traditional ORB-SLAM2 system can only construct sparse feature point maps for camera localization and cannot provide the environmental geometric information required for robot spatial inference and navigation. For this purpose, DMSG-SLAM incorporates a truncated signed distance field reconstruction module for dynamic environments. Figure 4 shows the pipeline for constructing the TSDF-based dense volumetric map. In order to achieve online mapping under limited computational resources, the updates of this module are entirely driven by the front-end keyframes of the system.

In the local dense point cloud extraction stage, the system adopts a conservative spatial masking strategy, directly removing all RGB-D data within potential dynamic target boxes in the new keyframe. The retained static background depth data is transformed into the world coordinate system and subjected to voxel downsampling and statistical outlier removal to generate a low-noise, local dense point cloud. Next, a global dense point cloud is formed through incremental fusion.

However, simply concatenating local point clouds can introduce severe data redundancy and thickness noise. To overcome this limitation, the system further introduces an Octree-based surface representation layer using TSDF. This module performs step sampling along the ray direction from the sensor’s optical center to the measurement point, calculates the normalized signed distance within the truncation distance, and uses a weighted moving average strategy to update the cumulative distance and confidence weight of each spatial voxel in real time. By leveraging a sparse voxel structure indexed by octree keys [35], the memory footprint of the global voxel grid is drastically reduced. Finally, the system extracts and publishes voxelized surface point clouds with visual textures by locating contour voxels that are close to zero in the distance field. This architecture reduces ineffective ray sampling operations and achieves smooth, continuous, and memory-efficient online dense geometric reconstruction in dynamic environments through the mechanism of keyframe triggering and dynamic region masking.

## 4. Experimental Results

This section presents a comprehensive evaluation of the proposed method using two widely adopted public benchmarks, namely the TUM RGB-D dataset [36] and the Bonn dataset [37]. These benchmarks include diverse dynamic scenarios, providing a suitable basis for performance validation. The evaluation is conducted using standard trajectory error metrics, including Absolute Trajectory Error (ATE) and Relative Pose Error (RPE), where RPE is further decomposed into Relative Translation Error (RTE) and Relative Rotation Error (RRE). To quantitatively assess accuracy, the Root Mean Square Error (RMSE) and Standard Deviation (S.D.) are employed. To demonstrate the effectiveness of the proposed approach, comparisons are first carried out against ORB-SLAM2 and several representative state-of-the-art dynamic SLAM methods on the selected datasets. Furthermore, ablation experiments are performed to analyze the contribution of each component in the proposed fusion framework. In addition, mapping quality is evaluated, followed by an investigation of the real-time performance of the system. All experiments are implemented on a computing platform running Ubuntu 20.04, equipped with an Intel i5-12490F processor (Intel, Santa Clara, CA, USA), an NVIDIA GeForce RTX 4060 GPU (Nvidia, Santa Clara, CA, USA), and 8 GB of memory. Unless otherwise specified, all key parameters were kept unchanged across the TUM and Bonn datasets. The main parameter settings used in all experiments are summarized in Table 1.

### 4.1. Evaluation on the TUM RGB-D Dataset

To assess the localization performance of the proposed system under dynamic conditions, five representative sequences are selected from the TUM RGB-D benchmark, including fr3_walking_xyz, fr3_walking_static, fr3_walking_rpy, fr3_walking_half, and fr3_sitting_static. These sequences are chosen due to their diverse motion patterns and varying levels of dynamic interference. The naming convention of the sequences encodes their characteristics: the terms “walking” and “sitting” describe the activity of the person present in the scene, while “xyz”, “rpy”, “half”, and “static” correspond to different camera motion configurations.

#### 4.1.1. Comparison with the Baseline Algorithm

As our system is a modification of the classical visual SLAM system ORB-SLAM2, we first conduct comparative experiments between our system and ORB-SLAM2. Table 2, Table 3 and Table 4 summarize the results on five dynamic TUM sequences based on ATE and RPE [38].

**Figure 5:**
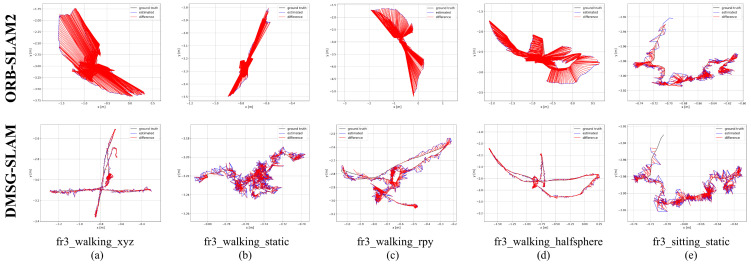
Schematic comparison of ATE results for DMSG-SLAM and ORB-SLAM2 across five dynamic sequences. (**a**) fr3_walking_xyz. (**b**) fr3_walking_static. (**c**) fr3_walking_rpy. (**d**) fr3_walking_halfsphere. (**e**) fr3_sitting_static.

**Figure 6:**
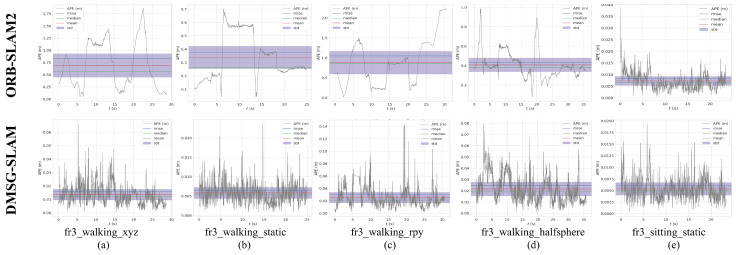
Comparison of Absolute Trajectory Error (ATE) trends between DMSG-SLAM and ORB-SLAM2 on five dynamic sequences. (**a**) fr3_walking_xyz, (**b**) fr3_walking_static, (**c**) fr3_walking_rpy, (**d**) fr3_walking_halfsphere, (**e**) fr3_sitting_static.

The experimental results in Table 2, Table 3 and Table 4 demonstrate that, across various sequences on the TUM dataset, our system achieves clear improvements over the baseline ORB-SLAM2 in terms of absolute trajectory error, translational drift error and rotational drift error. Comparing the four ATE metrics (RMSE, Mean, Median, S.D.), DMSG-SLAM shows a clear improvement over the baseline on highly dynamic sequences, with an average improvement of 90%. On the low-dynamic sequence, however, as the movements of a person seated in a chair are relatively small, the impact on localization is minimal, resulting in a less pronounced improvement of 20% on average. For intuitive comparison, Figure 5 presents the ATE curves of five representative sequences. The ground truth, estimated trajectory, and corresponding error are shown in black, blue, and red, respectively, with the red region indicating the error magnitude [39]. In high-motion sequences, DMSG-SLAM yields noticeably smaller errors than ORB-SLAM2, while in low-motion sequences it maintains comparable performance. Figure 6 further shows that DMSG-SLAM exhibits reduced ATE fluctuations. Consistent with Table 3 and Table 4, these results suggest that the proposed dynamic feature pruning strategy improves localization accuracy and reduces both translational and rotational drift in dynamic environments.

#### 4.1.2. Comparison with Advanced Algorithms

To further assess the performance of the proposed method, comparative evaluations are carried out against several existing approaches, such as DS-SLAM [40], YOLO-SLAM [41], SG-SLAM [42], NGD-SLAM, and RTS-SLAM, on five dynamic TUM sequences. The results are shown in Table 5. The best results are highlighted in bold. For the fr3_walking_static and fr3_sitting_static sequences, the camera undergoes only limited motion, so motion blur is negligible; the mask filters out a large number of dynamic feature points, and the remaining dynamic features near object boundaries are further removed by geometric constraints. In the fr3_walking_rpy sequence, RTS-SLAM employs a multi-constraint dynamic feature rejection strategy that integrates semantic information, geometric constraints, and trajectory consistency analysis. The combination of these constraints may help alleviate the influence of dynamic features under large viewpoint variations, resulting in relatively better localization performance. Although our method does not achieve the lowest error on this sequence, it attains the second-best performance among all compared methods, indicating its effectiveness in handling challenging rotational motions and dynamic disturbances. For the fr3_walking_xyz sequence, the significant translational motion of the camera generates sufficient parallax. By combining object detection with the Depth-RANSAC method, YOLO-SLAM effectively removes dynamic features while preserving static features within the detected regions, thereby achieving more accurate trajectory estimation. For the fr3_walking_half sequence, NGD-SLAM employs a more precise object detection algorithm, thereby providing a more continuous mask and more effectively filtering out dynamic feature points.

### 4.2. Evaluation on the BONN Dataset

To further validate the algorithm’s generalization capability across different dynamic environments, the Bonn RGB-D dataset was incorporated into the experiments. Released in 2019, this dataset comprises 24 highly dynamic indoor sequences. We selected nine representative challenging scenarios for evaluation: ‘Crowd’ features multiple people moving erratically indoors; ‘Moving non-box’ involves scenarios where pedestrians carry objects; ‘Person tracking’ simulates continuous tracking of moving targets by a camera; and ‘Synchronous’ depicts vigorous, coordinated jumping movements by multiple people. Compared with the TUM dataset, the Bonn sequences impose more intense and complex dynamic disturbances, providing a challenging benchmark for evaluating the effectiveness of dynamic feature rejection in SLAM systems.

Table 6 presents the evaluation results on the Bonn dataset, where we compare the baseline ORB-SLAM2 with several state-of-the-art algorithms, including SG-SLAM, NGD-SLAM, YOLO-SLAM, and RTS-SLAM. In the crowd2 sequence, when multiple human figures overlap across consecutive frames, the lightweight object detection algorithm may fail to detect some objects. Although geometric verification is used as a fallback, certain dynamic feature points may still be involved in localization and tracking. NGD-SLAM alleviates this issue by employing a more accurate object detection algorithm. In the Moving non-box sequence, our method may retain some feature points on the pedestrian’s feet due to the limited depth difference between the feet and the ground. NGD-SLAM’s hybrid feature point tracking strategy helps alleviate this issue; although some feature points may still be retained during operation, the number is likely to be smaller than in our approach. In the person_tracking2 sequence, RTS-SLAM further removes residual dynamic feature points that are not completely filtered out by geometric constraints by evaluating the consistency between feature point trajectories and the estimated camera motion across consecutive frames. In other highly dynamic sequences, our method shows competitive performance. The dynamic mask suppresses the most prominent dynamic interference, while the rotation-adaptive epipolar geometric constraints further filter out residual dynamic points. Furthermore, as the camera moves irregularly during tracking, the rotation-adaptive mechanism tends to discard ambiguous feature points more conservatively, thereby reducing the likelihood of retaining features on moving objects. Overall, the proposed method demonstrates competitive generalization capability across most highly dynamic sequences.

### 4.3. Ablation Experiment

Within the ablation analysis, DMSG-SLAM(S) is defined as a variant that discards feature points located inside the bounding regions of potentially dynamic objects. DMSG-SLAM (G) refers to the removal of dynamic points using only epipolar geometry. DMSG-SLAM (DM) is a method that uses only dynamic masking to remove dynamic feature points. DMSG-SLAM (S+G) is a method that utilizes semantic information to assist epipolar constraints in removing dynamic feature points, while DMSG-SLAM (DM+S+G) is a combined version of dynamic masking and semantic-guided epipolar geometric constraints. Finally, DMSG-SLAM (DM+S+GC) combines dynamic masking with rotation-adaptive semantic-guided epipolar geometric constraints, representing the final version of the algorithm. We conducted ablation experiments on five dynamic sequences from the TUM dataset, performing comparative analysis using two metrics from ATE. As shown in Table 7, whether through object detection, epipolar geometric constraints, or depth masking, all methods are capable of pruning dynamic objects to some extent, thereby improving the algorithm’s localization accuracy in dynamic environments. The depth mask serves, to some extent, as an enhanced version of the bounding box pruning method; it retains more usable static feature points within the bounding box, thereby improving localization accuracy. The incorporation of semantic information into the epipolar geometric constraints introduces a hierarchical pruning criterion, indicating which areas warrant particular attention. As the depth mask is not particularly refined, it struggles to cover all moving objects under rapid camera rotation; therefore, semantic-guided epipolar geometric constraints are introduced following the depth mask to further process moving feature points within the target bounding box. The inclusion of rotation-adaptive semantic-guided epipolar geometric constraints helps alleviate the depth mask’s shortcoming of incomplete masking during rapid rotation, enhancing the system’s localization capability by further filtering out moving points. As shown in Table 7, DMSG-SLAM (DM+S+GC) achieved the highest accuracy on four of the five sequences in the experiments. In the fr3_walking_xyz sequence, the false removal of some static points affected the localization accuracy; however, the method remains effective in most scenarios.

**Figure 7:**
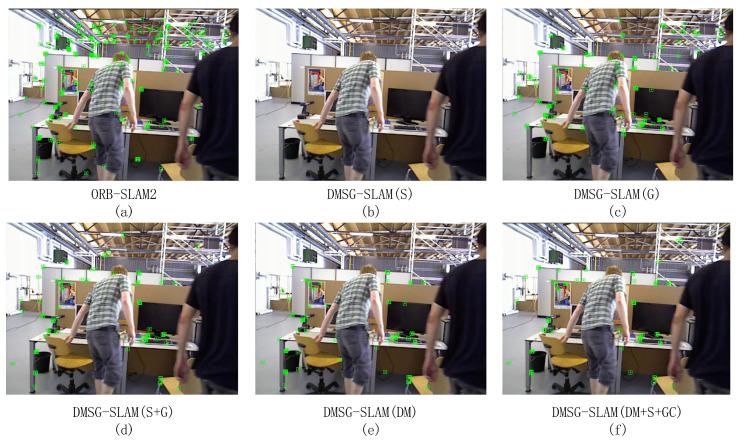
Qualitative comparison of dynamic feature rejection.Green points indicate the matched feature points after dynamic feature removal. (**a**) ORB-SLAM2. (**b**) DMSG-SLAM (S). (**c**) DMSG-SLAM (G). (**d**) DMSG-SLAM (S+G). (**e**) DMSG-SLAM (DM). (**f**) DMSG-SLAM (DM+S+GC).

As shown in Figure 7a, the rendered image of ORB-SLAM2 at the current frame contains a large number of feature points located on the moving person. Figure 7b shows the result of removing dynamic feature points using only bounding boxes. No feature points are plotted in the figure because the bounding boxes occupy too much space, resulting in too few matchable static feature points and consequently causing the tracking to fail. Figure 7c shows the result of removing dynamic feature points using only epipolar geometric constraints. By applying a fixed threshold for removal, the number of feature points on the moving object has decreased compared to Figure 7a. Figure 7d shows the result of removal using semantic-geometric constraints. By setting different thresholds for feature points inside and outside the bounding box, it can be seen that the performance is improved; however, some dynamic points are still matched. Figure 7e shows the results of the masking-only method, with almost no feature points remaining on the moving person, confirming its effectiveness. However, dynamic points remain along the edges of the person and on the chair. As the person is currently in the act of pulling the chair and the chair is also in motion, the feature points on the chair should have been removed; the masking method alone struggles to handle this situation. Figure 7f shows the results of combining dynamic depth masking with rotation-adaptive semantic-geometric constraints. It can be seen that no feature points are retained on the moving object, and the elimination is more accurate and refined. Figure 7 and Table 7 together demonstrate the effectiveness of the DMSG-SLAM fusion algorithm.

**Figure 8:**
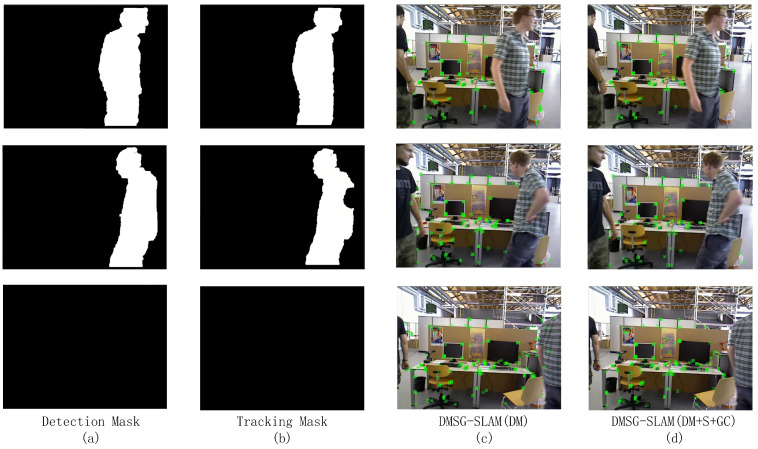
Validity verification of the out-of-detection-box geometric constraint.Green points indicate matched feature points. (**a**) Detection Mask. (**b**) Tracking Mask. (**c**) DMSG-SLAM (DM). (**d**) DMSG-SLAM (DM+S+GC).

When addressing missed detections of dynamic objects near image boundaries, the proposed out-of-mask fallback mechanism may provide certain benefits. As illustrated in Figure 8, a pedestrian is partially located outside the image frame, which can cause the object detector to fail to recognize the corresponding region. Consequently, no effective mask is generated in either the semantic detection thread or the tracking thread, as shown in the first and second subfigures of Figure 8. Under such circumstances, methods that rely solely on masks may incorrectly treat the pedestrian as part of the static background, as depicted in the third subfigure of Figure 8. In contrast, the proposed approach introduces a global epipolar geometric verification as a complementary strategy. This process tends to filter out feature points whose deviations exceed a predefined threshold of 1.0 pixel, thereby helping identify and suppress potentially undetected dynamic features, as illustrated in the fourth subfigure of Figure 8. Overall, this mechanism can partially alleviate the limitations of the detection network in such scenarios.

**Figure 9:**
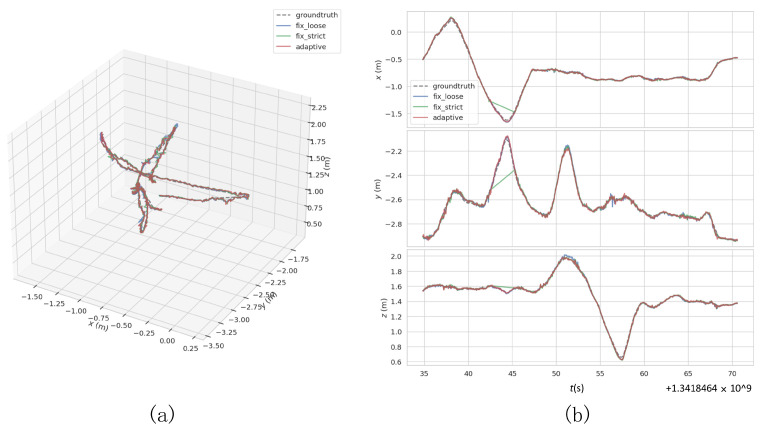
The 3D trajectories for different epipolar geometry threshold strategies and their XYZ component curves over time in fr3_walking_half. (**a**) 3D trajectory (**b**) Curves showing changes in the X, Y and Z axes over time.

Figure 9 presents the 3D trajectories and the corresponding XYZ axis-wise temporal curves under different epipolar threshold strategies on the challenging fr3_walking_half sequence. To evaluate the necessity of the adaptive strategy, comparisons are made with two fixed-threshold variants. The rotation threshold for triggering adaptation is set to $\theta_{\mathrm{th}}=0.015\;\mathrm{rad}$. The fixed strict threshold uses a constant epipolar distance of $\tau_{\mathrm{strict}}=0.5$ pixels, while the fixed loose threshold adopts $\tau_{\mathrm{loose}}=0.7$ pixels. As shown in the axis-wise curves on the right side of Figure 9, the fixed strict threshold causes trajectory discontinuity between 42 s and 46 s, since the overly strict criterion removes too many static background points during slight camera rotations. In contrast, the fixed loose threshold maintains continuity but fails to suppress some dynamic features during stronger motion, resulting in noticeable local drift along the Y-axis and a final ATE RMSE of 0.0266 m. By comparison, the rotation-adaptive strategy reduces the RMSE to 0.0248 m by preserving tracking continuity under stable motion and tightening the threshold within potential dynamic regions under increased rotation.

To further examine the generality of this observation, Table 8 presents the results of the fixed-loose, fixed-strict, and adaptive strategies on representative TUM sequences. In most cases, the adaptive strategy achieves lower or similar RMSE compared with the two fixed-threshold settings while maintaining relatively stable variation. These results suggest that the proposed switching strategy offers a reasonable balance between dynamic feature removal and static feature retention under different motion conditions. Nevertheless, the adaptive criterion should still be regarded as a heuristic switching strategy based on estimated camera rotation rather than a theoretically optimal rule. Its practical effect may vary with the reliability of motion estimation, the selected thresholds, and the complexity of scene dynamics.

**Figure 10:**
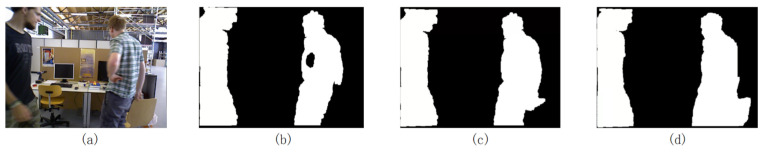
Detector Mask refinement results under different depth consistency thresholds $\tau_{d}$ on a representative dynamic frame. (**a**) the input RGB image, (**b**) the refined mask with $\tau_{d}=0.25$ m, (**c**) the refined mask with $\tau_{d}=0.33$ m, (**d**) the refined mask with $\tau_{d}=0.40$ m.

Table 9 and Figure 10 jointly illustrate the effect of the depth consistency threshold $\tau_{d}$. Quantitatively, $\tau_{d}=0.25$ m is associated with higher RMSE and a larger standard deviation on both representative sequences, suggesting that an overly strict depth tolerance may reduce stability. In contrast, $\tau_{d}=0.33$ m achieves the lowest error on fr3_walking_static and the same RMSE as $\tau_{d}=0.40$ m on fr3_walking_half, while yielding a lower standard deviation. Qualitatively, Figure 10 shows that a smaller threshold tends to cause boundary shrinkage, whereas a larger threshold tends to produce thicker masks and include more nearby non-target regions. Overall, $\tau_{d}=0.33$ m appears to provide a more balanced trade-off between boundary completeness and background suppression, and is therefore selected as the default setting.

Table 10 reports the sensitivity analysis of different $\tau_{\mathrm{strict}}/\tau_{\mathrm{loose}}$ settings. It can be observed that the overall performance varies only moderately under reasonable perturbations, indicating that the proposed adaptive strategy is not overly sensitive to this parameter pair. Compared with the stricter setting (0.4/0.6) and the looser setting (0.6/0.8), the default configuration (0.5/0.7) achieves lower or comparable RMSE on most representative sequences while showing a more balanced standard deviation. Therefore, 0.5/0.7 is adopted as the default setting to provide a more reasonable trade-off between dynamic feature suppression and static correspondence preservation.

### 4.4. Analysis of Mapping Results

**Figure 11:**
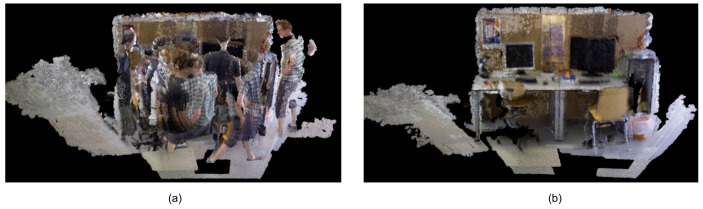
Dense point cloud reconstruction results on the TUM dataset. (**a**) Dense map of fr3_walking_xyz without removing dynamic objects. (**b**) Dense map of fr3_walking_xyz after removing dynamic objects.

Experiments are carried out on the TUM RGB-D benchmark to evaluate mapping performance. Two forms of map representation are considered for visualization, including a traditional dense point cloud and a TSDF-based volumetric reconstruction. A qualitative comparison on the fr3_walking_xyz sequence is presented in Figure 11. As shown in Figure 11a, when dynamic object removal is not applied, the reconstructed map contains noticeable artifacts introduced by moving human elements, which adversely affect the overall quality. By contrast, the result in Figure 11b demonstrates that, after filtering out dynamic objects, the generated dense map becomes cleaner, with reduced interference from transient structures.

**Figure 12:**
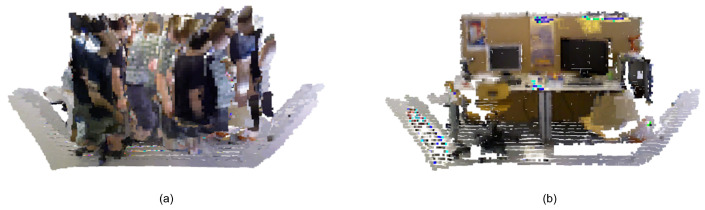
TSDF-based dense volumetric mapping results on the TUM dataset. (**a**) TSDF map of the fr3_walking_static sequence without dynamic object removal. (**b**) TSDF map of the fr3_walking_static sequence with dynamic objects removed.

Figure 12 shows a surface representation layer based on a truncated signed distance field. Figure 12a and Figure 12b illustrate the mapping results after retaining and removing moving objects, respectively. A comparison of the two maps reveals that Figure 12b successfully removes moving people while effectively capturing the geometric details of the tabletop. Compared with point cloud maps, the surface representation layer based on truncated signed distance fields produces a smoother, continuous surface mesh. To objectively characterize the computational overhead of the mapping module, the average TSDF fusion time is approximately 13.6 ms per frame. For the TSDF result shown in Figure 12b, the final reconstruction contains 15,175 extracted surface points and 75,149 active TSDF voxels. In brief, the surface point count reflects the scale of the final visible surface, while the active TSDF voxel count reflects the spatial extent of the TSDF region involved in fusion updates. Although our DMSG-SLAM successfully removes extensive dynamic ghosting, slight color bleeding and texture blurring can still be observed on the reconstructed TSDF surface. This artifact manifests as erroneous colors from subsequent frames being slightly superimposed onto the static background. This phenomenon stems from multi-view color misalignment. During the incremental TSDF fusion process, the tracking thread inevitably experiences millimeter-scale trajectory jitter, leading to pixel-to-voxel projection shifts.

### 4.5. Time Analysis

To evaluate the real-time performance of the DMSG-SLAM algorithm, we recorded the average tracking time per frame and compared it with several state-of-the-art systems. As shown in Table 11, on our hardware platform, DMSG-SLAM achieved an average processing time of 25.32 ms. This performance meets the real-time requirement of a 30 fps RGB-D camera. Although our system introduces a slight computational overhead of approximately 5 ms compared with the baseline ORB-SLAM2 algorithm, this is an acceptable trade-off. This additional processing time is primarily attributed to the semantic masking and rotation-adaptive epipolar geometric culling modules. By incurring minimal computational overhead, DMSG-SLAM efficiently filters out dynamic interference while retaining static feature points within the target region.

## 5. Conclusions

This paper presents DMSG-SLAM, an RGB-D SLAM system designed for complex dynamic environments. By employing a sequential strategy of “mask-based pre-filtering with geometric fallback,” the system effectively addresses the issue of incomplete semantic boundary coverage, while the rotation-adaptive geometric constraints improve tracking stability under challenging motion conditions. Experimental evaluations on the TUM and Bonn datasets demonstrate that DMSG-SLAM achieves more accurate pose estimation and clearer Octree-based TSDF surface reconstruction across most dynamic scenarios.

However, the proposed framework may still lead to the inadvertent removal of some static features, and several geometric thresholds currently require manual tuning. In addition, the adaptive criterion remains heuristic in nature, and its effectiveness may depend on motion estimation accuracy, threshold selection, and scene dynamics. Moreover, although the framework demonstrates practical effectiveness on the experimental platform, its computational cost in embedded or edge-computing scenarios deserves further investigation. In particular, the object detection module constitutes the dominant runtime overhead, whereas the optical flow, DBSCAN clustering, and geometric filtering stages are relatively lightweight. Future work will focus on automatic parameter adaptation, model compression, platform-specific optimization for resource-constrained deployment, and delayed color fusion for more realistic static dense reconstruction.

## Figures and Tables

**Table 1:** Unified key parameter settings used in all experiments.

Parameter	Value	Unit	Description
$\tau_{d}$	0.33	m	Depth consistency threshold used for local mask refinement.
ϵ	50	px	DBSCAN radius used for clustering motion-consistent points.
$\tau_{\mathrm{strict}}$	0.5	px	Stricter epipolar threshold used inside potential dynamic target boxes under larger inter-frame rotation.
$\tau_{\mathrm{loose}}$	0.7	px	Looser epipolar threshold used inside potential dynamic target boxes under smaller inter-frame rotation.
$\tau_{\mathrm{out}}$	1.0	px	Fixed epipolar threshold used for feature points outside potential dynamic target boxes.
$\theta_{\mathrm{th}}$	0.015	rad	Rotation threshold for switching between $\tau_{\mathrm{strict}}$ and $\tau_{\mathrm{loose}}$.

**Table 2:** Comparison of the Absolute Trajectory Error (ATE) between DMSG-SLAM and ORB-SLAM2 on the TUM dataset (Unit: m).

Sequences	ORB-SLAM2				DMSG-SLAM (Ours)				Improvements			
	RMSE	Mean	Median	S.D.	RMSE	Mean	Median	S.D.	RMSE (%)	Mean (%)	Median (%)	S.D. (%)
fr3_walking_xyz	0.8382	0.6888	0.5639	0.4775	0.0158	0.0135	0.0123	0.0081	98.12	98.04	97.83	98.31
fr3_walking_static	0.3779	0.3382	0.2638	0.1685	0.0065	0.0058	0.0055	0.0029	98.28	98.28	97.93	98.27
fr3_walking_rpy	1.0486	0.8785	0.8517	0.5725	0.0305	0.0252	0.0213	0.0171	97.09	97.13	97.50	97.01
fr3_walking_half	0.4312	0.4075	0.3828	0.1411	0.0248	0.0216	0.0189	0.0124	94.24	94.71	95.05	91.24
fr3_sitting_static	0.0083	0.0073	0.0066	0.0039	0.0061	0.0053	0.0048	0.0030	26.32	27.43	28.02	22.63

**Table 3:** Comparison of the Relative Position Error (RPE) between DMSG-SLAM and ORB-SLAM2 on the TUM dataset (Unit: m/s).

Sequences	ORB-SLAM2				DMSG-SLAM (Ours)				Improvements			
	RMSE	Mean	Median	S.D.	RMSE	Mean	Median	S.D.	RMSE (%)	Mean (%)	Median (%)	S.D. (%)
fr3_walking_xyz	0.4373	0.3690	0.3108	0.2346	0.0183	0.0164	0.0150	0.0082	95.82	95.57	95.16	96.52
fr3_walking_static	0.2011	0.0867	0.0137	0.1814	0.0070	0.0064	0.0067	0.0029	96.51	92.64	51.25	98.39
fr3_walking_rpy	0.4308	0.2800	0.1060	0.3274	0.0383	0.0334	0.0321	0.0188	91.10	88.06	69.70	94.26
fr3_walking_half	0.3192	0.1737	0.0512	0.2678	0.0254	0.0207	0.0194	0.0147	92.05	88.09	62.09	94.50
fr3_sitting_static	0.0093	0.0081	0.0073	0.0047	0.0068	0.0060	0.0055	0.0032	27.30	25.79	24.47	32.07

**Table 4:** Comparison of the Rotation Drift Error (RPE) between DMSG-SLAM and ORB-SLAM2 on the TUM dataset (Unit: °/s).

Sequences	ORB-SLAM2				DMSG-SLAM (Ours)				Improvements			
	RMSE	Mean	Median	S.D.	RMSE	Mean	Median	S.D.	RMSE (%)	Mean (%)	Median (%)	S.D. (%)
fr3_walking_xyz	7.2504	5.9103	4.2613	4.1996	0.4629	0.4260	0.4003	0.1812	93.62	92.79	90.61	95.68
fr3_walking_static	3.5476	1.5458	0.3106	3.1931	0.2162	0.1863	0.1750	0.1098	93.90	87.95	43.66	96.56
fr3_walking_rpy	7.8776	5.2512	2.8034	5.8721	0.7914	0.7277	0.7035	0.3111	89.95	86.14	74.91	94.70
fr3_walking_half	6.3058	3.5596	1.0346	5.2051	0.6789	0.5925	0.5246	0.3316	89.23	83.36	49.29	93.63
fr3_sitting_static	0.2944	0.2648	0.2518	0.1286	0.2444	0.2215	0.2007	0.1034	16.97	16.37	20.29	19.56

**Table 5:** Comparison of the absolute trajectory error (ATE) between the method proposed in this paper and state-of-the-art methods on the TUM dataset (Unit: m).

Seq.	DS-SLAM		YOLO-SLAM		SG-SLAM		NGD-SLAM		RTS-SLAM		DMSG-SLAM (Ours)	
	RMSE	S.D.	RMSE	S.D.	RMSE	S.D.	RMSE	S.D.	RMSE	S.D.	RMSE	S.D.
fr3_w_xyz	0.0247	0.0161	**0.0146**	**0.0070**	0.0171	0.0088	0.0151	0.0075	0.0162	0.0084	0.0158	0.0081
fr3_w_static	0.0081	0.0036	0.0073	0.0035	0.0079	0.0036	0.0071	0.0036	0.0068	0.0032	**0.0065**	**0.0029**
fr3_w_rpy	0.4442	0.2350	0.2164	0.1001	0.0316	0.0181	0.0334	0.0189	**0.0263**	**0.0165**	0.0305	0.0171
fr3_w_half	0.0303	0.0159	0.0283	0.0138	0.0305	0.0163	**0.0245**	**0.0119**	**0.0245**	0.0129	0.0248	0.0124
fr3_s_static	0.0065	0.0033	0.0066	0.0033	0.0087	0.0045	0.0066	**0.0030**	0.0068	0.0039	**0.0061**	**0.0030**

*Note:* Bold values indicate the best results.

**Table 6:** Comparison of the absolute trajectory error (ATE) between the method proposed in this paper and state-of-the-art methods on the Bonn dataset (Unit: m).

Seq.	ORB-SLAM2		SG-SLAM		NGD-SLAM		YOLO-SLAM		RTS-SLAM		DMSG-SLAM (Ours)	
	RMSE	S.D.	RMSE	S.D.	RMSE	S.D.	RMSE	S.D.	RMSE	S.D.	RMSE	S.D.
crowd1	0.6215	0.3777	0.0217	0.0119	0.0223	0.0143	0.033	–	0.0210	0.0117	**0.0177**	**0.0091**
crowd2	1.1349	0.4322	0.0584	0.0406	**0.0213**	**0.0120**	0.423	–	0.0246	0.0143	0.0267	0.0175
crowd3	1.0639	0.4698	0.0592	0.0350	0.0332	0.0236	0.069	–	0.0309	0.0181	**0.0278**	**0.0188**
moving_no_box1	0.1196	0.0908	0.0283	0.0171	**0.0195**	**0.0063**	0.027	–	0.0211	0.0122	0.0197	0.0071
moving_no_box2	0.0479	0.0173	0.0299	0.0105	0.0316	0.0107	0.035	–	0.0268	0.0104	**0.0242**	**0.0092**
person_tracking1	0.6422	0.2981	0.0417	0.0110	0.0462	0.0125	0.157	–	0.0382	0.0115	**0.0372**	**0.0137**
person_tracking2	0.7778	0.4731	0.0376	0.0154	0.0617	0.0162	0.037	–	**0.0251**	0.0131	0.0373	**0.0114**
synchronous1	0.8188	0.4447	0.3229	0.1824	0.0204	0.0166	0.014	–	0.0822	0.0564	**0.0130**	**0.0080**
synchronous2	1.3350	0.1639	0.0164	0.0126	0.0091	0.0041	0.007	–	0.0138	0.0088	**0.0067**	**0.0036**

*Note:* Bold values indicate the best results.

**Table 7:** ATE comparison of different modules on the TUM dataset (Unit: m).

Seq.	DMSG-SLAM (S)		DMSG-SLAM (G)		DMSG-SLAM (DM)		DMSG-SLAM (S+G)		DMSG-SLAM (DM+S+G)		DMSG-SLAM (DM+S+GC)	
	RMSE	S.D.	RMSE	S.D.	RMSE	S.D.	RMSE	S.D.	RMSE	S.D.	RMSE	S.D.
fr3_w_xyz	0.0171	0.0088	0.0174	0.0091	**0.0153**	**0.0077**	0.0165	0.0085	0.0157	0.0084	0.0158	0.0081
fr3_w_static	0.0092	0.0050	0.0110	0.0072	0.0081	0.0046	0.0076	0.0035	0.0069	0.0034	**0.0065**	**0.0029**
fr3_w_rpy	0.373	0.0262	0.0358	0.0246	0.0402	0.0270	0.0306	0.0184	0.0316	0.0172	**0.0305**	**0.0171**
fr3_w_half	0.0364	0.0234	0.0366	0.0201	0.0282	0.0142	0.0303	0.0165	0.0266	0.0157	**0.0248**	**0.0124**
fr3_s_static	0.0088	0.0044	0.0077	0.0034	0.0069	0.0032	0.0076	0.0039	0.0066	0.0032	**0.0061**	**0.0030**

*Note:* Bold values indicate the best results.

**Table 8:** Comparison of Epipolar Thresholding Strategies.

Sequence	Fixed-Loose (τ=0.7)		Fixed-Strict (τ=0.5)		Adaptive (0.7/0.5)	
	RMSE	S.D.	RMSE	S.D.	RMSE	S.D.
fr3_w_xyz	0.0157	0.0084	0.0163	0.0084	0.0158	0.0081
fr3_w_static	0.0069	0.0034	0.0069	0.0032	0.0065	0.0029
fr3_w_rpy	0.0316	0.0172	0.0309	0.0179	0.0305	0.0171
fr3_w_half	0.0266	0.0157	0.0254	0.0131	0.0248	0.0124
fr3_s_static	0.0066	0.0032	0.0065	0.0033	0.0061	0.0030

**Table 9:** Sensitivity analysis of the depth consistency threshold $\tau_{d}$ on representative TUM sequences.

$\tau_{d}$	fr3_walking_half		fr3_walking_static	
	RMSE	S.D.	RMSE	S.D.
0.25	0.0287	0.0149	0.0085	0.0056
0.33	0.0282	0.0142	0.0081	0.0046
0.40	0.0282	0.0149	0.0082	0.0046

**Table 10:** Sensitivity analysis of different $\tau_{\mathrm{strict}}/\tau_{\mathrm{loose}}$ settings on representative TUM sequences.

Sequence	0.4/0.6		0.5/0.7		0.6/0.8	
	RMSE	S.D.	RMSE	S.D.	RMSE	S.D.
fr3_w_xyz	0.0161	0.0082	0.0158	0.0081	0.0159	0.0078
fr3_w_static	0.0066	0.0030	0.0065	0.0029	0.0067	0.0033
fr3_w_half	0.0265	0.0142	0.0248	0.0124	0.0246	0.0128

**Table 11:** Average processing time per frame for different systems.

Systems	Average Processing Time per Frame (ms)	Hardware Platform
ORB-SLAM2	20.18	Intel i5-12490F CPU, NVIDIA GeForce GTX 4060 GPU
SG-SLAM	23.78	Intel i5-12490F CPU, NVIDIA GeForce GTX 4060 GPU
NGD-SLAM	17.86	Intel i5-12490F CPU, NVIDIA GeForce GTX 4060 GPU
RTS-SLAM	24.25	Intel i5-12490F CPU, NVIDIA GeForce GTX 4060 GPU
DMSG-SLAM (ours)	25.32	Intel i5-12490F CPU, NVIDIA GeForce GTX 4060 GPU

## Data Availability

The original contributions presented in this study are included in the article. Further inquiries can be directed to the corresponding author.
